# Estimated incidence of cardiovascular complications related to type 2 diabetes in Mexico using the UKPDS outcome model and a population-based survey

**DOI:** 10.1186/1475-2840-10-1

**Published:** 2011-01-07

**Authors:** Nancy Reynoso-Noverón, Roopa Mehta, Paloma Almeda-Valdes, Rosalba Rojas-Martinez, Salvador Villalpando, Mauricio Hernández-Ávila, Carlos A Aguilar-Salinas

**Affiliations:** 1 Oficina del Subsecretario de Salud, Secretaria de Salud,(Lieja 7, Colonia Juárez), México City (06600) México; 2 Departamento de Endocrinología y Metabolism, Instituto Nacional de Ciencias Médicas y Nutrición "Salvador Zubirán", (Vasco de Quiroga 15, Colonia Tlalpan), Mexico City (14000), México; 3 Centro de Investigación en Salud Poblacional, Instituto Nacional de Salud Publica, (Universidad 655, Colonia Santa María Ahuacatitlán), Cuernavaca, Morelos (62100), México

## Abstract

**Background:**

To estimate the incidence of complications, life expectancy and diabetes related mortality in the Mexican diabetic population over the next two decades using data from a nation-wide, population based survey and the United Kingdom Prospective Diabetes Study (UKPDS) outcome model

**Methods:**

The cohort included all patients with type 2 diabetes evaluated during the National Health and Nutrition Survey (ENSANut) 2006. ENSANut is a probabilistic multistage stratified survey whose aim was to measure the prevalence of chronic diseases. A total of 47,152 households were visited. Results are shown stratified by gender, time since diagnosis (> or ≤ to 10 years) and age at the time of diagnosis (> or ≤ 40 years).

**Results:**

The prevalence of diabetes in our cohort was 14.4%. The predicted 20 year-incidence for chronic complications per 1000 individuals are: ischemic heart disease 112, myocardial infarction 260, heart failure 113, stroke 101, and amputation 62. Furthermore, 539 per 1000 patients will have a diabetes-related premature death. The average life expectancy for the diabetic population is 10.9 years (95%CI 10.7-11.2); this decreases to 8.3 years after adjusting for quality of life (CI95% 8.1-8.5). Male sex and cases diagnosed after age 40 have the highest risk for developing at least one major complication during the next 20 years.

**Conclusions:**

Based on the current clinical profile of Mexican patients with diabetes, the burden of disease related complications will be tremendous over the next two decades.

## Background

Diabetes is the principal cause of death in Mexico [1]. Its prevalence in adults over the age of 20 has grown from 6.7% in 1993 to nearly 14% in 2006 [2-4]. Nationwide population based surveys providing unbiased information regarding the prevalence and clinical characteristics of persons with diabetes are scant [5,6]. In Mexico, three nationwide surveys have shown that adults with type 2 diabetes have a high prevalence of co-morbid conditions which contribute to the high incidence of macrovascular and microvascular complications. Regrettably, the incidence of chronic complications is unknown.

Simulation modeling is gaining acceptance as a valuable tool for providing long-term information regarding prognosis; such information is often unavailable from clinical studies. Several simulation models [7-12] are available for estimating the incidence of diabetic complications. The United Kingdom Prospective Diabetes Study (UKPDS) outcome model is the most popular. This model was developed using data obtained from patients who participated in the UKPDS. In this study the medical history, biochemical variables and diabetes related complications were documented in the vast majority of patients, with very few patients lost during follow-up [13,14].

Since predictions using validated tools and population representative data have not been published in Latin American countries, the objective of this report is to estimate the incidence of complications, life expectancy, quality-adjusted life expectancy and diabetes related mortality in the Mexican diabetic population over the next two decades. This will allow us to simulate the challenges that the Mexican health system will face in the next 20 years.

## Materials and methods

### Population sample

The 2006 National Health and Nutrition Survey (ENSANut) was a cross sectional study including individuals representative of those living in Metropolitan, urban and rural areas. A multistage, stratified and probabilistic sampling procedure was used to collect information. A random sample of Basic Geographical Statistical Units was obtained in all states of Mexico, and neighborhood blocks were randomly selected. In every home, a randomly selected adult, adolescent, child and health service user were invited to participate. A target of 4731 individuals and 1476 households was estimated per state. The total number of households visited was 48600. A sample of this size is capable of detecting risk factors that have a state-wide prevalence of at least 8.1% with a relative error of estimation of 0.25, a design effect of 1.7 and a non-response rate of 20%. The study was carried out in accordance with the Helsinki Declaration of Human Studies. Informed consent was obtained from each participant. A separate consent form was signed by participants who provided blood samples. The study was approved by the Research and Ethics committees of the Instituto Nacional de Salud Pública.

### Methods

The procedures of the study are reported in detail elsewere [15,16]. Briefly, a structured interview was conducted and a previously standardized questionnaire was used to obtain demographic, socioeconomic, family health history, past medical history and lifestyle information (e.g. smoking). Diabetes was considered present if subjects referred to a previous diagnosis or if the fasting plasma glucose was ≥ 126 mg/dl [14]. Type 1 diabetes was diagnosed if insulin treatment was required during the first two months after the diagnosis or if the patient had history of ketoacidosis. Fasting blood samples were obtained in approximately 30% of the adult population (n = 6613). These cases were randomly distributed among study subjects. This subsample has a statistical power to detect conditions with a nation-wide prevalence ≥ 8%. The response rate was 85%. Detailed information about the methods used for the collection of the data is published elsewhere [15]. The socio-demographic parameters (age, gender, socioeconomic status and body mass index) of the subsample were not different from the rest of the population. The sampling procedure was standardized during a two week training course. The subjects were sampled at their homes; they remained seated for five minutes before the blood sample was drawn. All analytical measurements were carried out in the laboratories of the Instituto Nacional de Salud Pública using commercial reagents described in detail elsewere [15].

### Simulation model

The United Kingdom Prospective Diabetes (UKPDS) outcome model was used to estimate life expectancy, quality-adjusted life years (QALY), risk for the development of fatal and non fatal myocardial infarction, ischemic heart disease, cerebrovascular event, cardiac failure, amputation and death in the diabetic population. The model was developed based on observations and follow up over 10 years of 5102 patients with newly diagnosed type 2 diabetes. It considers four risk factors (HbA1c, HDL-cholesterol, total cholesterol, systolic blood pressure and smoking) as longitudinal data and adjusts a model of random effects to estimate the pattern over time. Equations for HbA1c, systolic blood pressure, total cholesterol and HDL-cholesterol are based on annual changes of each risk factor, while change in smoking habit is calculated in three-year time periods from the diagnosis of diabetes. With respect to diabetic complications, Weibull proportional risks regression model was used to estimate occurrence of a compound result for fatal and non-fatal events. In this simulation model, ischemic heart disease and congestive heath failure events are registered only if they occurred prior to a myocardial infarction event. Separate equations were used to model diabetes and diabetes related mortality using a combination of Gompertz regression equations and logistic regression models. The impact of different complications was obtained through the EQ-5D health condition questionnaire given to patients free of complications. It is assumed that multiple complications have an additive effect on quality of life. The simulations are carried out in annual cycles. A combination of bootstrap methods and multiple attribution were used to handle uncertainty, such that confidence intervals reflect uncertainty of the parameter in the model [14]. Missing data were attributed considering the mean values of the conditions according to age (< 40, 40-60 and > 60 years), gender and time of diagnosis (< 10 and > 10 years). A license was obtained for the use of the UKPDS outcome model (Serial 1896, July 21,2005). The UKPDS Results Model version 1.1 was run with 1000 Monte Carlo essays per subject and with 10 re-samplings (bootstraps) to handle uncertainty. It was assumed that clinical variables remained unchanged over time. The model's three ethnic groups do not include the Mexican population, therefore, the group most closely related to our study population was selected (Asian Indians). Native American and Asian populations share peculiarities in their body composition. In these populations, the mean body mass index (BMI) and height are lower than that observed for Caucasians, although the tendency towards abdominal obesity may be greater [17,18]. Life expectancy, quality-adjusted life expectancy, expected incidence of chronic complications were calculated for a 20 year period. Results are presented by gender, time of diagnosis (> or ≤ to 10 years) and age of diagnosis (> or ≤ 40 years).

### Validation of the UKPDS outcome model for cardiovascular (CVD) events in Mexicans

The UKPDS outcome model has not been applied previously in Mexicans. As a consequence, we run a separate study to validate the estimates. For this purpose, a retrospective study based on the medical charts of patients with type 2 diabetes treated for at least 10 years at the Instituto Nacional de Ciencias Médicas y Nutrición was performed. Patients lost to follow up for more than one year were excluded. Predicted (using baseline variables at diagnosis) and observed proportions of primary cardiovascular events were compared. The discrimination (c-statistic) and calibration (Hosmer-Lemeshow χ^2^) of the UKPDS outcome model were calculated. A total of 1089 records were reviewed. Of these 654 records were excluded for the following reasons: 35 patients with type 1 diabetes mellitus, 13 patients with secondary diabetes, 379 without HbA1c results, 42 without a lipid profile, 2 patients were younger than age 20 at the time of diagnosis and 182 patients had cardiovascular disease diagnosed in the initial visit. Thus, the study sample was composed of 436 patients (217 men and 219 women). The mean age at diagnosis of diabetes was 48.7 years. One hundred and one (23.1%) died during the follow-up period. Cardiovascular disease was the cause of death in 52 patients (51.4%). In addition, 260 cardiovascular events were recorded (coronary events = 45, stroke = 45). Discrimination of the UKPDS outcome model for CVD events was 0.66 (IC 95% 0.59-0.72). The calibration was poor (Hosmer-Lemeshow χ^2 ^= 23.8, p = 0.03), but of the same magnitude as that reported by Guzder [14]. The sensitivity and specificity for an estimated 10-year CVD risk of 20% was 89% and 36%, respectively.

## Results

There were 6613 subjects in our study over the age of 20, representing 56,745,719 Mexicans. Seven percent of the subjects had previous knowledge of their diabetic condition. However, on analysis of biological samples, the actual prevalence of diabetes was 14.4%. All cases were considered as having type 2 diabetes; no case with type 1 diabetes was identified. If we extrapolate this figure to the general population, the actual number of Mexicans with diabetes is 7,316,950. The clinical characteristics of the diabetic population are shown in Table 1.

**Table 1:** Description of the diabetic population included in the Encuesta Nacional de Salud y Nutrición 2006

Population of 20 years or older	n = 56,745,719
**Diabetic population**	

Previously diagnosed	3,138,720 (7.0)

Diagnosed during the survey	4,178,230 (7.4%)

Total	7,316,950 (14.4)

**Time from diagnosis (years)**	0.5(0-7)

**Age (years)**	52 (42-62)

Men	51(41-60)

Women	53 (42-64)

**Weight(kg)**	71.6 (64-80.8)

**Height (m)**	1.57 (1.5-1.63)

**Total Cholesterol (mmol/l)**	5.22 (5.14-5.29)

**HDL cholesterol (mmol/l)**	1.02 (1.01-1.03)

**Systolic blood pressure (mmHg.)**	130 (120-140)

**HbA1c (%)**	11.4 (10.15-11.8)

**Smoking**	

Never	5,395,934 (68.4)

Past	1,174,763(14.9)

Currently	1,322,280(16.8)

Data are shown either as median (interquartile range) or number (%)

### Twenty year incidence of macrovascular complications

We estimate that 112 (95% CI 84-140) per 1000 persons with diabetes will present at least one ischemic heart disease event, corresponding to 882,433 calculated events (95% CI 661,744-1,103,123). In addition, we estimate that 113 (95% CI 65-161) per 1000 persons with diabetes will develop a heart failure event, resulting in 889,443 new events (95% CI 509,638-1,269,248). With respect to myocardial infarction, we calculate that 260 (95% CI 215-304) per 1000 diabetic patients will develop this condition. This would correspond to 2,048,996 (95% CI 1,699,743-2,398,248) new cardiovascular events. With respect to the incidence of cerebral vascular disease, we estimate that 101 (95% CI 69-133) per 1000 persons with diabetes will have this type of complication (798,188 new cases (95% CI 544,809-1,051,568)). Finally, 62 (95% CI 40-85) per 1000 persons with diabetes will present an amputation; corresponding to 491,236 (95% CI 313,900-668,572) amputations. These predicted incidences are presented in Table 2.

**Table 2:** Expected incidence of cardiovascular complications in persons with diabetes in Mexico

Simulated years	Accumulated probability	Number of expected cases/1000 diabetics	Confidence Interval 95%		Total expected cases (CI95%)
Ischaemic heart disease					

1	0.009	9	7	12	73,482 (52,026-94,939)

5	0.039	39	29	49	311,146 (232,355-389,936)

10	0.069	69	53	86	547,679 (415,306-680,052)

15	0.094	93	71	116	738,373 (557,617-919,129)

20	0.112	112	84	140	882,433 (661,744-1,103,123)

Myocardial infarction					

1	0.019	20	13	26	155,025 (102,215-207,836)

5	0.088	88	67	109	695,535 (526,932-864,139)

10	0.159	159	128	191	1,258,903 (1,010,065-1,507,742)

15	0.216	217	178	255	1,710,383 (1,405,667-2,015,099)

20	0.259	260	215	304	2,048,996 (1,699,743-2,398,248)

Heart failure					

1	0.010	10	5	16	81,640 (35,674-127,607)

5	0.039	40	22	57	311,887 (177,573-446-201)

10	0.069	70	41	98	551,131 (325,182-777,081)

15	0.094	94	55	133	742,646 (434,411-1,050,081)

20	0.112	113	65	161	889,443 (509,638-1,269-248)

Cerebrovascular disease					

1	0.008	9	4	13	68,070 (35,505-100,636)

5	0.035	36	24	48	282,893 (186,677-379,110)

10	0.063	63	44	82	498,794 (348,475-649,112)

15	0.085	85	59	111	671,080 (469,221-872,938)

20	0.101	101	69	133	798,188 (544,809-1,051,568)

Amputation					

1	0.006	7	2	11	51,666 (16,656-86,676)

5	0.021	22	12	31	170,582 (94,289-246,875)

10	0.036	36	23	49	282,205 (179,425-384,985)

15	0.049	49	32	66	389,413 (255,649-523,177)

20	0.062	62	40	85	491,236 (313,900-668,572)

Differences between Mexicans and the ethnic groups studied in the UKPDS study could add uncertainty to the results provided by the outcome model. Based on that, we re-ran the estimates in order to measure the magnitude of the ethnicity effect among the options provided by the simulation model. As shown in table 3, Asian ethnicity renders a slightly higher mean 20 years probability for having ischemic heart disease, myocardial infarction and death compared to the Caucasian background. However, none of these differences reached statistical significance. The differences are significantly greater when compared against the Afro-Caribbean heritage. However, African heritage is very small (< 5%) in the Mexicans [19]. Thus, the Afro-Caribbean ethnicity is not a valid option for our study sample. In summary, the selection of the Asian Indians as the group most closely related to our study population may have minor consequences on the estimates provided by the UKPDS outcomes model.

**Table 3:** Twenty year accumulated probability for having macrovascular complications or death: effect of ethnicity

Outcome	Asian	White	Afro-Caribbean
Ischemic heart disease	0.112 (0.084-0.14)	0.090 (0.064-0.117)	0.096 (0.068-0.124)

Myocardial infarction	0.259 (0.215-0.304)	0.220 (0.174-0.265)	0.075* (0.015-0.134)

Heart failure	0.112 (0.065-0.161)	0.115 (0.067-0.163)	0.124 (0.072-0.175)

Cerebrovascular disease	0.101 (0.069-0.133)	0.096 (0.067-0.125)	0.110 (0.080-0.140)

Amputation	0.062 (0.040-0.085)	0.063 (0.040-0.086)	0.068 (0.043-0.093)

Death	0.538 (0.508-0.570)	0.529 (0.495-0.563)	0.489 (0.455-0.524)

Data are presented as means (95% confidence intervals)

* p < 0.001 AfroCaribbean versus Asian or White

### Death and life expectancy

The model estimated that 539 (CI95% 508-570) per 1000 diabetic persons will have died within 20 years. In 2005, the Mexican National Statistics and Geography Institute (INEGI) estimated an average life expectancy of 74.6 years in the general population [20]. For individuals with diabetes, the UKPDS model predicts an average life expectancy (LE) of 10.9 years (95%CI 10.7-11.2); this decreases to 8.3 years (95%CI 8.1-8.5) when QALY are considered. Hence the average life expectancy for persons with diabetes is 62.9 years. On adjustment for quality of life, this figure is reduced to 60.3 years (see table 4).

**Table 4:** Expected mortality in persons with diabetes in Mexico

Simulated years	Accumulated probability	Number of expected cases/1000 persons with diabetes	95% Confidence Interval		Total expected cases (95% CI)
1	0.043	43	33	54	345,066 (263,705-426,428)

5	0.162	162	144	180	1,280,852 (1,138,935-1,422,769)

10	0.292	292	272	312	2,305,420 (2,148,055-2,462,786)

15	0.416	416	394	440	3,290,559 (3,111,409-3,469,410)

20	0.538	538	508	570	4,251,383 (4,007,547-4,495,220)

Finally, when analyzing for gender, disease duration and age at diagnosis, men older than 40 years at the time of diagnosis and men with more than 10 years of diabetes are expected to have a higher incidence of complications (table 5). The life expectancy was greater for women (11.1 years, 95% CI 10.8-11.4) compared to men (10.7 years, 95% CI 10.5-11). The same was true for patients aged 40 or younger (12.8 years, 95% CI 12.6-13.0) compared to older subjects (10.1 years, 95% CI 9.8-10.3).

**Table 5:** Expected twenty years incidence of cardiovascular complications and life expectancy by gender, time of evolution and age of diagnosis

Complication	Probability (95% CI)	Expected Cases (95% CI)	Probability (95% CI)	Expected Cases (95% CI)	Probability (CI95%)	Expected Cases* (CI95%)
	**Women** **n = 3,931,127**		**Time evolution** **≤ 10 years** **n = 6,652,964**		**Age at diagnosis** **≤ 40 years** **n = 2,590,066**	

**Ischemic heart**	0.095 (0.066-0.124)	373,457 (259,454-487,460)	0.117 (0.089-0.145)	778,397 (592,114-964,680)	0.087 (0.060-0.115)	225,336 (155,404-297,858)

**Myocardial infarction**	0.196 (0.142-0.249)	770,501 (558,220-978,851)	0.264 (0.223-0.305)	1,756,382 (1,483,611-2,029,154)	0.129 (0.096-0.162)	334,119 (248,646-419,591)

**Heart failure**	0.127 (0.072-0.183)	499,253 (283,041-719,396)	0.107 (0.065-0.148)	711,867 (432,443-984,639)	0.037 (0.016-0.058)	95,832 (41,441-150,224)

**Cerebrovascular disease**	0.093 (0.063-0.122)	365,595 (247,661-479,597)	0.097 (0.068-0.126)	645,338 (452,402-838,273)	0.030 (0.003-0.057)	77,702 (7,770-147,634)

**Amputation**	0.065 (0.040-0.089)	255,523 (157,245-349,870)	0.058 (0.040-0.077)	385,872 (266,119-512,278)	0.065 (0.036-0.094)	168,354 (93,242-243,466)

**Death**	0.531 (0.498-0.565)	2,087,428 (1,957,701-2,221,087)	0.467 (0.435-0.499)	3,106,934 (2,894,039-3,319,829)	0.283 (0.250-0.317)	7,332,989 (647,517-821,051)

	**Men** **n = 3,961,850**		**Time evolution** **> 10 years** **n = 1,240,012**		**Age at diagnosis** **> 40 years** **n = 5,302,911**	

**Ischemic heart disease**	0.136 (0.102-0.170)	538,812 (404,109-673,515)	0.080 (0.050-0.110)	99,201 (62,001-136,401)	0.122 (0.091-0.152)	646,955 (482,565-806,042)

**Myocardial infarction**	0.352 (0.315-0.390)	1,394,571 (1,247,983-1,545,122)	0.231 (0.161-0.301)	286,443 (199,642-372,244)	0.315 (0.260-0.370)	1,670,417 (1,378,757-1,962,077)

**Heart failure**	0.091 (0.053-0.128)	360,528 (209,978-507,117)	0.148 (0.056-0.241)	183,522 (69,441-298,843)	0.145 (0.085-0.204)	768,922 (450,747-1,081,794)

**Cerebrovascular disease**	0.112 (0.074-0.149)	443,727 (293,177-590,316)	0.120 (0.065-0.175)	148,801 (80,601-217,002)	0.131 (0.094-0.168)	694,681 (498,474-890,889)

**Amputation**	0.059 (0.039-0.079)	233,749 (154,512-312,986)	0.088 (0.037-0.139)	109,121 (45,880-172,362)	0.062 (0.041-0.082)	328,780 (217,419-434,839)

**Death**	0.550 (0.522-0.578)	2,179,018 (2,068,086-2,289,949)	0.956 (0.928-0.984)	1,185,451 (1,150,731-1,220,172)	0.647 (0.612-0.681)	3,430,983 (3,245,382-3,611,282)

## Discussion

The results of three population-based nationwide surveys have shown that the prevalence of type 2 diabetes has grown rapidly in México over the past few decades. More than seven million Mexican adults now live with diabetes. This report clearly shows that the burden imposed by diabetes to Mexico will be very high risk over the next twenty years. This report makes emphasis in macrovascular complications, the main cause of death among patients with type 2 diabetes [21]. The UKPDS outcome model estimates that 53.9% (95 CI% 50.8-57%) of currently affected subjects will be dead by the year 2026. Their life expectancy will be reduced to an average of 10.9 years (IC95% 10.7-11.2). The predicted 20 year-incidence of the principal cardiovascular complications per 1000 diabetic individuals are: ischemic heart disease 112, myocardial infarction 260, heart failure 113, stroke 101 and amputation 62. These predictions must urge the Mexican health system to establish effective treatment programs and improve diabetes care. In the absence of such measures, the resources required to manage future diabetes related complications will surpass the capability of the Mexican health system.

### Strengths of the study and implications for the Mexican Health system

**Figure 1:**
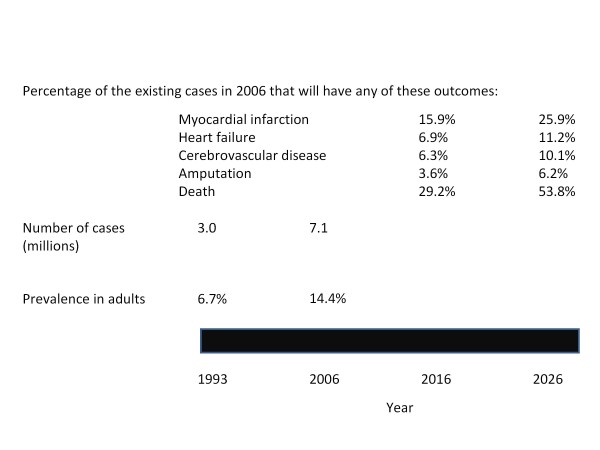
**Current and future burden imposed by type 2 diabetes in Mexico**.

Simulation modeling is gaining acceptance as a valuable tool for providing long-term prognostic information [22]. Such data is often unavailable from clinical or epidemiological studies. Its use has been incorporated in the analyses of several multicenter trials [23] and cost effectiveness studies [24]. The validity of the estimates depends on the model's assumptions and on the quality of data selected as inputs for the program. The UKPDS outcomes model is one of the more accepted simulation models, because it is based on prospectively registered data [13]. This risk engine performs better than the Framingham equation for the prediction of coronary heart disease rates [25]. With regards to the quality of data, we have used the best possible source of information to make our estimates. Data was obtained from a population based, nationwide survey and collected using a probabilistic, multistaged, stratified sampling method. This guarantees national, regional and urban/rural representation. Trained personnel obtained information using standardized methodologies. Also, estimates were done for special groups, such as cases diagnosed before age 40. In countries, like México, in which diabetes is highly prevalent, the onset of the disease happens at earlier ages [26]. This feature boosts the adverse consequences of diabetes [27]. The longer exposure to hyperglycemia and other diabetes-related abnormalities increase the likelihood that patients will develop chronic complications. Thus, our data provides a panorama of possible scenarios regarding the incidence of diabetic complications that Mexico may face in the near future. Even if we consider only the most conservative estimates, the future looks bleak (figure 1). Systematic analyses of institutional registers will be required to validate the precision of the estimates reported here.

### Limitations of the study

The main disadvantage of simulation models is that estimates are not precise for persons who have characteristics different to that of persons who participated in the original trial. Latino populations, for example, were not included in the UKPDS. Despite this concern, the UKPDS outcome model has been applied in multiethnic study samples [28,29]. To counter this factor, we treated our study sample as Asian Indians, which is an ethnic group included in the UKPDS study that shares biological and socio-economical characteristics with Latin populations [17,18]. We re-ran the estimates in order to measure the magnitude of the ethnicity effect among the options provided by the simulation model. Minor, non significant differences were found if an Asian or a Caucasian background was assumed in the modeling process. Thus, the selection of the Asian Indians as the group most closely related to our study population may have minor consequences on the estimates reported here. In addition, we validated the prognostic value for CVD events of the UKPDS outcome model in a group of Mexican patients followed up for over ten years. The discrimination (c = 0.66 (IC 95% 0.59-0.72) for CVD events reported here is similar to that reported in the multiethnic ADVANCE study (c = 0.70 (0.65-0.76)) [28] and in a cohort composed of Caucasians (c = 0.670) [14]. This observation suggests that the UKPDS outcome model estimates may be as valid in Mexicans as in other ethnic groups. Other limitations may affect our ability to predict the CVD outcomes. The UKPDS outcome model measures the probability of presenting the first event, but not subsequent ones. For instance, it does not consider the existence of a double amputation. Also, the model simulates the progression of diabetes under "conventional treatment" within the UKPDS study. Conventional treatment in Mexico is much more varied. In addition, the UKPDS risk engines apply to people with newly diagnosed diabetes. Here, the model was applied to both undiagnosed and diagnosed cases of diabetes. Finally, the estimates are derived from a single measurement. Despite these limitations, the value of the present analysis depends largely on the extent to which the participants of the Encuesta Nacional de Salud y Nutricion are representative of current populations of patients with type 2 diabetes living in México.

The UPKDS outcome model is thought to either overestimate [24] or underestimate CVD risk [14] depending on the nature of the population under study. It is probable that the model may overestimate the CVD risk in a population-based sample [30] like the one reported here. Our CVD estimates are similar to those reported in Mexican-Americans from the 1999-2002 National Health and Nutrition Examination Survey [29]. The 10 year risk of CVD events (ischemic heart disease plus myocardial infarction) estimated in our sample (22.8%) was almost identical (22.5%) to that found in the Mexican Americans.

## Conclusions

The impact of type 2 diabetes on the Mexican health system will be significantly greater in the next two decades. Simulation modeling shows that if the clinical characteristics of the diabetic population remain unaltered, a large proportion of the diabetic population will suffer premature mortality and disabilities in the coming years.

## Competing interests

The authors declare that they have no competing interests.

## Authors' contributions

NR-N. researched data and wrote manuscript. RM. reviewed/edited manuscript. PA-V researched data. RR-M researched data, reviewed/edited manuscript. SV. researched data. MH-A contributed to discussion, reviewed/edited manuscript. CAA-S. researched data and wrote manuscript. All authors read and approved the final manuscript.
